# Reply to: Heliox in the treatment of status asthmaticus: case reports

**DOI:** 10.5935/0103-507X.20160060

**Published:** 2016

**Authors:** Inês Carvalho, Sara Querido, Joana Silvestre, Pedro Póvoa

**Affiliations:** Polyvalent Intensive Care Unit, Hospital de São Francisco Xavier, Centro Hospitalar de Lisboa Ocidental - Lisbon, Portugal.; Chronic Disease Studies Center, Faculdade de Ciências Médicas, Universidade Nova de Lisboa - Lisbon, Portugal.

Dr. Chantar et al.'s letter to the Editor raises three relevant issues on various aspects of mechanical ventilation in status asthmaticus.

In regard to the first point, we agree with the comment. The potential benefit of helium results from its lower density and the implications that this brings to fluid mechanics.

The second issue raises several questions. Regarding the accuracy of volume measurement using the Maquet Servo-i ventilator with Heliox, we confess that we did not perform bench tests. However, the equipment has European certification (CE) and is also certified by the Food and Drug Administration (FDA). We used a pneumatic system for the aerosols, which generates particles of <5 µm at a flow rate of 8L/m gas (air/O_2_). Lastly, we used a heat/moisture exchanger humidification system.

The last issue is the most important and refers to ventilator parameterization in severe asthma.^(1-3)^ The first comment refers to the I:E ratio. Perhaps there was misinterpretation of the data. In the Maquet Servo-i ventilator, the titration of the recommended inspiratory flow, 60 - 70L/m, is calculated using the setting of tidal volume (Vt), respiratory rate, and I:E ratio; programming was always carried out with the intent of shortening inspiratory time and increasing expiratory time. The respiratory rate was kept purposely low in accordance with recommendations,^(1-3)^ as this is the only way to increase expiratory time, even if at the expense of hypoventilation.^(3)^ It has been well-demonstrated that respiratory rate has a marked influence on end-expiratory flow and dynamic hyperinflation.^(4)^ The recommended Vt is 7 - 9mL/kg;^(1,3)^ in our patients (#1: 60 kg; #2: 70kg), we initially used a Vt of 6mL/kg; subsequently, following their clinical improvement, the Vt could be increased.

The issue of positive end-expiratory pressure (PEEP) merits a specific approach. Zero PEEP is not "antiphysiological", as there is no physiological PEEP. In healthy individuals, end-expiratory alveolar pressure is equal to atmospheric pressure. Only patients with an obstructive disease, acute asthma or chronic obstructive pulmonary disease, have dynamic hyperinflation and positive end-expiratory alveolar pressure.^(5)^ In invasive mechanical ventilation, extrinsic PEEP (PEEPe) has three indications:^(3)^ reduction of respiratory work in patients with intrinsic PEEP (PEEPi) when on assisted ventilation, hypoxemic respiratory failure due to pulmonary edema/atelectasis, and prevention of ventilator-associated pneumonia.

Finally, there is the matter of zero end-expiratory pressure (ZEEP). The effect of PEEPe on dynamic hyperinflation and PEEPi depends on its physiopathological mechanism.^(2,3)^ In patients without expiratory flow limitation, such as in the case of asthma, PEEPe is entirely transmitted to the distal airways, leading to increased alveolar pressure.^(2,3)^ Application of PEEPe in patients with severe asthma is therefore harmful and not recommended.^(1,3)^ Finally, we come to the shunt issue. There are two aspects to this issue. Patients with severe asthma may develop atelectasis caused by secretion plugs; in this case, its resolution is better achieved using bronchoscopy rather than by applying PEEPe. The hypoxemia mechanism acts by changing the ventilation perfusion ratio rather than by shunt. Another mechanism is severe hyperinflation is a consequence of the compression of the pulmonary capillaries and a large increase in dead space. In our patients, ZEEP was only applied during controlled ventilation, so the question of respiratory work did not arise.

Inês Carvalho and Sara Querido

Polyvalent Intensive Care Unit, Hospital de São Francisco Xavier, Centro Hospitalar de Lisboa Ocidental - Lisbon, Portugal.

Joana Silvestre and Pedro Póvoa

Polyvalent Intensive Care Unit, Hospital de São Francisco Xavier, Centro Hospitalar de Lisboa Ocidental - Lisbon, Portugal and Chronic Disease Studies Center, Faculdade de Ciências Médicas, Universidade Nova de Lisboa - Lisbon, Portugal.
